# A Shark Liver Gene-Derived Active Peptide Expressed in the Silkworm, *Bombyx mori*: Preliminary Studies for Oral Administration of the Recombinant Protein

**DOI:** 10.3390/md11051492

**Published:** 2013-05-07

**Authors:** Yunlong Liu, Ying Chen, Jianqing Chen, Wenping Zhang, Qing Sheng, Jian Chen, Wei Yu, Zuoming Nie, Yaozhou Zhang, Wutong Wu, Lisha Wang, Inthrani Raja Indran, Jun Li, Lian Qian, Zhengbing Lv

**Affiliations:** 1 Zhejiang Provincial Key Laboratory of Silkworm Bioreactor and Biomedicine, Institute of Biochemistry, Zhejiang Sci-Tech University, Hangzhou 310018, China; E-Mails: liuyunlong5566@163.com (Y.L.); carolynchency@163.com (Y.C.); cjqgqj@126.com (J.C.); zwpcc@126.com (W.Z.); csheng@zstu.edu.cn (Q.S.); chj1999@126.com (J.C.); mikkyu@163.com (W.Y.); wuxinzm@zstu.edu.cn (Z.N.); yaozhou@zstu.edu.cn (Y.Z.); wuwutong@gmail.com (W.W.); 2 School of Pharmacy, Xuzhou Medical College, Xuzhou 221004, China; E-Mail: gawlsv@163.com; 3 Department of Obstetrics & Gynaecology, Yong Loo Lin School of Medicine, National University Health System, Singapore 119228, Singapore; E-Mails: obgiri@nus.edu.sg (I.R.I.); jun_li@nuhs.edu.sg (J.L.); 4 Agilent Technologies Singapore Pte Ltd., Singapore 117681, Singapore; E-Mail: lovelyqianlian@gmail.com

**Keywords:** active peptide from shark liver, *Bombyx mori* pupae, BmNPV/Bac-to-Bac baculovirus expression system, type 2 diabetes mellitus, oral administration

## Abstract

Active peptide from shark liver (APSL) is a cytokine from *Chiloscyllium plagiosum* that can stimulate liver regeneration and protects the pancreas. To study the effect of orally administered recombinant APSL (rAPSL) on an animal model of type 2 diabetes mellitus, the APSL gene was cloned, and APSL was expressed in *Bombyx mori* N cells (BmN cells), silkworm larvae and silkworm pupae using the silkworm baculovirus expression vector system (BEVS). It was demonstrated that rAPSL was able to significantly reduce the blood glucose level in mice with type 2 diabetes induced by streptozotocin. The analysis of paraffin sections of mouse pancreatic tissues revealed that rAPSL could effectively protect mouse islets from streptozotocin-induced lesions. Compared with the powder prepared from normal silkworm pupae, the powder prepared from pupae expressing rAPSL exhibited greater protective effects, and these results suggest that rAPSL has potential uses as an oral drug for the treatment of diabetes mellitus in the future.

## 1. Introduction

Diabetes mellitus is a chronic metabolic disease characterized by increasing glucose blood levels. It is caused by the interaction of both genetic and environmental factors [1]. Type 2 diabetes mellitus is characterized by inadequate insulin secretion and insulin resistance (IR) and causes serious harm to humans, often leading to metabolic disorders involving carbohydrates, proteins and fats [1,2]. These disorders can result in serious complications involving blood vessels, nerves, the heart, kidneys and other organs. Currently, 366 million people suffer from diabetes mellitus, and an additional 280 million people have an identifiably high risk of developing diabetes. By 2030, the number of people with type 2 diabetes is expected to increase to 552 million, with an additional 398 million people at high risk [2]. Recently, diabetes treatment research has focused on identifying active compounds with hypoglycemic effects that act through novel mechanisms. Oral administration has been demonstrated to be a good delivery method for protein drugs. Zhang *et al.* showed that a protein expressed by a silkworm pupae bioreactor could act as an active cytokine when orally administered [3].

*Bombyx mori* (*B. mori*) has a well-studied genetic background and a highly synchronized development. Ryu *et al*. first reported that silkworm powder (prepared by lyophilization) had a positive effect on diabetic patients [4,5], and the blood glucose-lowering effect was verified by subsequent research [6]. Because *B. mori* is susceptible to infection by nuclear polyhedrosis virus and because it is easy to breed on a large scale, Maeda *et al*. (1985) utilized the *B. mori* nuclear polyhedrosis virus (BmNPV) system as an innovative tool for heterologous protein expression [7]. *B. mori* has been used as a bioreactor for the production of recombinant proteins using the BmNPV expression system [8]. Baculoviruses do not infect vertebrate animals, and the system itself is safe [9,10]. These features make the silkworm system an ideal expression and delivery package for producing medicinal proteins for oral administration [11,12]. A major advantage of the BmNPV expression system is that it can be used to produce relatively large quantities of post-translationally modified heterologous proteins [13]. This expression system is inexpensive, convenient and produces large amounts of proteins. This system has been widely used to express recombinant proteins [14]. 

Active peptide from shark liver (APSL) is an active protein that was initially purified from shark (*Chiloscyllium plagiosum*) liver [15,16,17]. APSL can improve the function of islet β cells and decrease the effect of streptozotocin on pancreatic β cells [18]. In addition, APSL can enhance the repair of damage to the pancreas and can even induce the pancreas to secrete insulin [19,20]. The functions of APSL indicate that it may have therapeutic effects on diabetes mellitus. However, it is difficult to obtain large amounts of naturally produced APSL. Therefore, it is important to obtain rAPSL through genetic engineering. In this study, a recombinant virus containing the *APSL* gene was produced using the Bac-to-Bac baculovirus expression system [21], and the rAPSL protein was expressed in *B. mori* silkworm larvae and pupae. This study may lay the basis for the treatment of diabetes mellitus using rAPSL.

## 2. Results

### 2.1. Construction of Recombinant Plasmid pFastBac HTA-APSL

**Figure 1:**
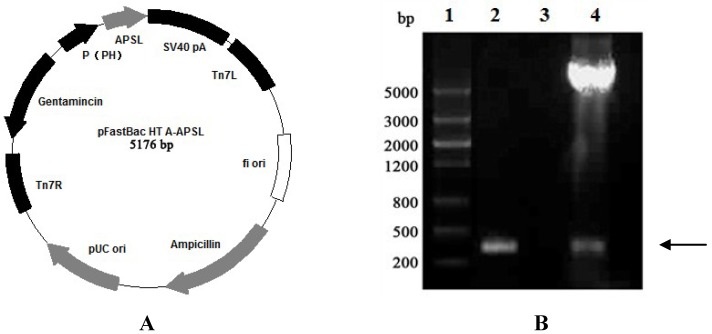
Identification of the recombinant plasmid (pFastBac HTA-*APSL*). The PCR product was electrophoresed on 1% agarose gel. (**A**) Recombinant plasmid analysis map. (**B**) Identification of the recombinant plasmid map. Lane 1: DNA Marker III (Shanghai Yuanye, China); Lane 2: PCR product of recombinant plasmid with P1/P2; Lane 3: PCR product of pFastBac HTA with P1/P2; Lane 4: double digest product (*Bam*H I/*Sal* I) of the recombinant plasmid.

To obtain the target *APSL* fragment, primers for polymerase chain reaction (PCR) amplification were synthesized, as described in the Materials and Methods. The *APSL* fragment (342 bp) was successfully produced from pET-28a-*APSL* using PCR. The *APSL* fragment was then ligated into the transfer vector, pFastBac HTA. Figure 1 shows the APSL fragments (marked by an arrow) obtained from the PCR and double digestion analyses in lanes 2 and 4, respectively. The fragment, which lies between 200 and 500 bp, is consistent with the expected size of the *APSL* fragment.

### 2.2. Construction and Isolation of Recombinant Bacmid

The recombinant donor plasmid pFastBac HTA-*APSL* was transformed into *E. coli* DH10Bac cells. After incubation, the white colonies, which are expected to represent recombinant BmNPV bacmids equipped with the *APSL* gene, were picked and cultured overnight in medium containing kanamycin, gentamycin and tetracycline. The recombinant bacmid obtained was named rBacmid-*APSL*.

**Figure 2:**
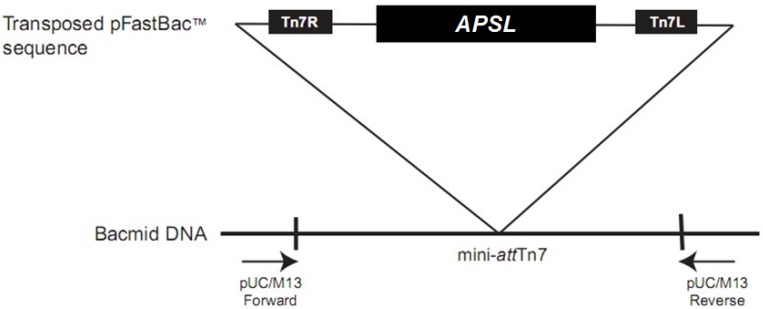
Transposition region analysis of pFastBac HTA-*ASPL*.

**Figure 3:**
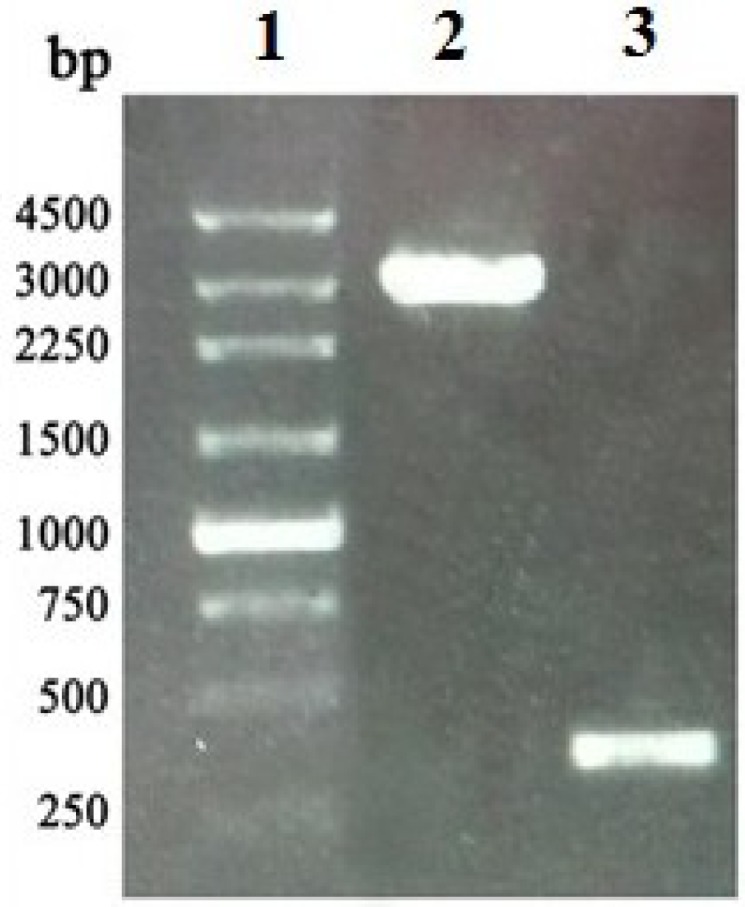
Identification of rBacmid-*APSL* by PCR. The PCR product was electrophoresed on 1% agarose gel. Lane 1: 250 bp DNA ladder marker; Lane 2: PCR product of rBacmid-*APSL* with M13 F/M13 R; Lane 3: negative control (PCR product of wild-type bacmid with M13 F/M13 R).

The rBacmid-*APSL* was isolated from the DH10Bac cells and identified by PCR with the M13 forward primer and the M13 reverse primer (Figure 2). Figure 3 shows a band at approximately 3000 bp (Lane 2), which is consistent with the 2436 bp plus the size of the target gene (342 bp).

### 2.3. Construction, Isolation and Analysis of Recombinant Bacmid

**Figure 4:**
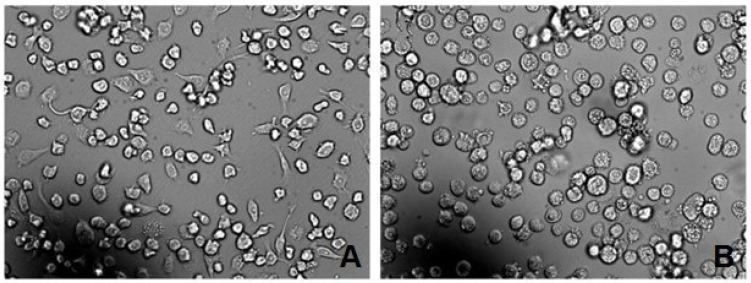
Microscopy images of different *Bombyx mori* N (BmN) cells. (**A**) BmN cells not transfected with rBacmid-*APSL* (20 × 10). (**B**) BmN cells transfected with rBacmid-*APSL* (20 × 10).

Following transfection of *Bombyx mori* N cells (BmN cells) with rBacmid-*APSL*, the cells became larger and rounded, and the refractive index of the cells increased. A number of BmN cells were in a suspended state (Figure 4). The recombinant virus (rBv-*APSL*) was generated in the transfected BmN cells after 3–5 days, and the morphological changes of transfected BmN cells were verified under an optical inverted microscope.

**Figure 5:**
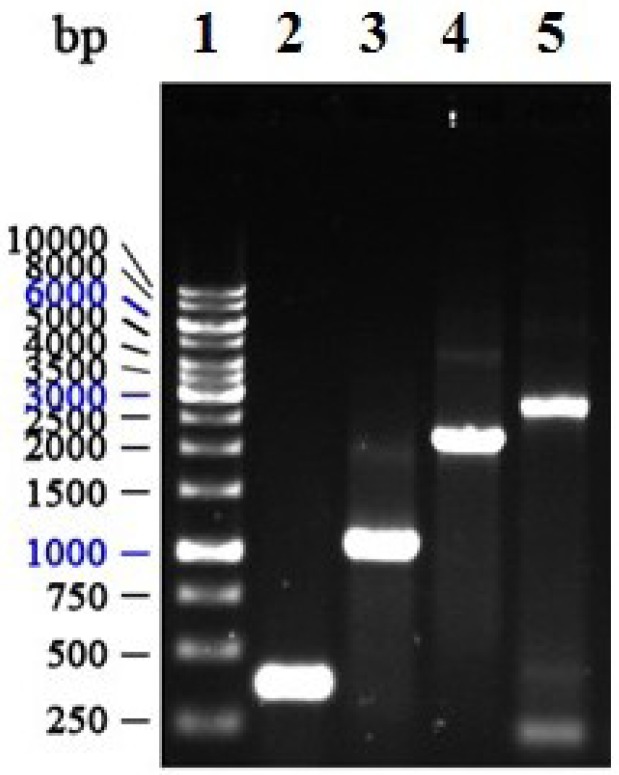
Identification of recombinant virus rBv-*APSL* DNA by PCR. The PCR product was electrophoresed on 1% agarose gel. Lane 1: GeneRuler™ 1 kb DNA Ladder; Lane 2: PCR product of rBv-APSL with P1/P2; Lane 3: PCR product of rBv-*APSL* with P1/M13 R; Lane 4: PCR product of rBv-*APSL* with M13 F/P2; Lane 5: PCR product of rBv-*APSL* with M13 F/M13 R.

Viral genomic DNA was extracted using a viral DNA purification kit, and the DNA was identified by PCR with the following primer pairs: P1 and P2, P1 and the M13 reverse primer, the M13 forward primer and P2 and the M13 forward and M13 reverse primers. The results of the PCR analysis, shown in Figure 5, are consistent with the expected results.

### 2.4. Expression, Collection and rAPSL Assay of BmN Cells

BmN cells (4 × 10^6^) were infected with the recombinant virus at a multiplicity of infection (MOI) of 10. The wild-type virus was used as the control. The infected cells were cultured, harvested and lysed. 

**Figure 6:**
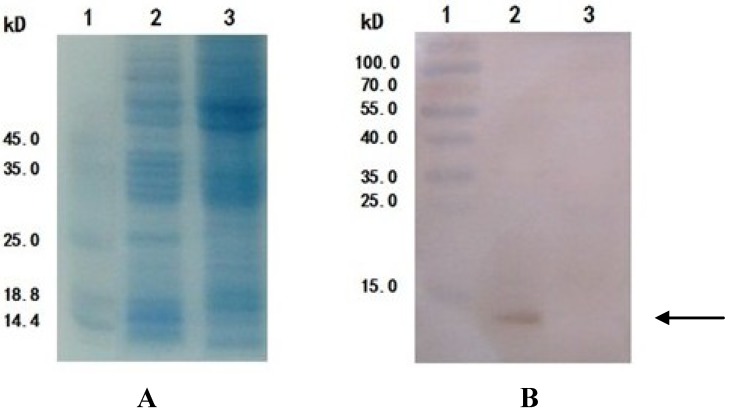
SDS-PAGE and Western blot analysis of the expressed rAPSL. (**A**) SDS-PAGE. Lane 1: protein molecular weight marker (low); Lane 2: BmN cells infected with rBv-*APSL*; Lane 3: BmN cells not infected with rBv-*APSL*. (**B**) Western blotting. Lane 1: pre-stained marker; Lane 2: BmN cells infected with rBv-*APSL*; Lane 3: BmN cells not infected with rBv-*APSL*.

Cells lysis supernatant from both rBv-*APSL* infected BmN cells and wild-type virus infected BmN cells were analyzed by Western blotting. A band was observed at approximately 15.0 kDa (Lane 2) after rBv-*APSL* infection in both the SDS-PAGE and Western blot analyses. No immunospeciﬁc signal corresponded in molecular mass to APSL in uninfected BmN cell samples (Figure 6).

### 2.5. Expression, Collection and rAPSL Assay of Silkworm Larvae and Pupae

**Figure 7:**
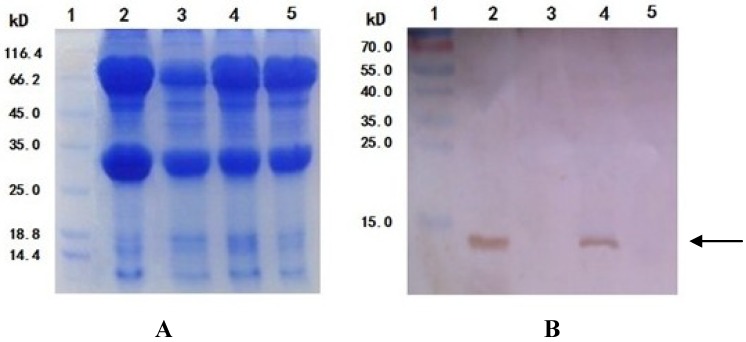
SDS-PAGE and Western blotting analysisof the expressed rAPSL. (**A**) SDS-PAGE. Lane 1: protein molecular weight marker (low); Lane 2: the pupal supernatant from silkworm pupae infected with rBv-*APSL*; Lane 3: the pupal supernatant from silkworm pupae not infected with rBv-*APSL*; Lane 4: the hemolymph from silkworm larvae infected with rBv-*APSL*; Lane 5: the hemolymph from silkworm larvae not infected with rBv-*APSL.* (**B**) Western blotting. Lane 1: pre-stained marker; Lane 2: the pupal supernatant from silkworm pupae infected with rBv-*APSL*; Lane 3: the pupal supernatant from silkworm pupae not infected with rBv-*APSL*; Lane 4: the hemolymph from silkworm larvae infected with rBv-*APSL*; Lane 5: the hemolymph from silkworm larvae not infected with rBv-*APSL.*

A solution of recombinant virus (MOI = 10) was injected into the body cavities of fifth instar larvae and diapausing pupae using a needle. The wild-type virus was used as a control. The infected silkworm larvae stopped feeding and crawled actively. The color of the hemolymph in the infected silkworm gradually changed from clear bright yellow to opaque dark yellow and finally to milky white. The body of the infected pupae gradually became soft and easy to brake and milky content overflowed. The hemolymph was collected from the silkworm larvae and pupae and stored at −80 °C until use. Larval hemolymph and pupal supernatant from both rBv-*APSL* infected silkworm and wild-type virus infected silkworm were diluted and separated by 12% SDS-PAGE, transferred to a NC ﬁlter membrane and analyzed by Western blotting. A band was observed at approximately 15.0 kDa (Lanes 2 and 4) after rBv-*APSL* infection in both the SDS-PAGE and Western blot analyses. No immunospeciﬁc signal corresponded in molecular mass to APSL in wild-type virus infected silkworm samples (Figure 7). 

### 2.6. Bioactivity Analysis of Silkworm Pupae-Expressed rAPSL

The levels of fasting blood glucose (FBG) measured before and after administration of rAPSL are shown in Table 1. The FBG level increased after the induction of streptozotocin (80 mg/kg body wt) and was maintained at approximately 25.6 ± 1.41 mmol/L in the fourth week, and all the animals in model group died during the fifth week. The FBG levels were significantly decreased to 15.00 ± 2.72 mmol/L (*P* < 0.001 *vs.* model) after the continuous administration of rAPSL for four weeks, indicating that rAPSL had protective effects against streptozotocin-induced diabetes mellitus by improving glucose metabolism in diabetic mice. In addition, it was observed that the normal silkworm pupae sample also exhibited a weak protective effect on streptozotocin-damaged islets (Figure 7).

**Table 1:** Effect of rAPSL on fasting blood glucose levels in diabetic mice.

Group	FBG (mmol/L)				
	First week	Second week	Third week	Forth week	Fifth week
Control	4.72 ± 0.92	4.81 ± 0.86	4.83 ± 1.07	4.83 ± 1.38	4.78 ± 1.16
Model	28.79 ± 3.44	27.28 ± 2.39	26.32 ± 3.07	25.6 ± 1.41 ^▲▲▲^	All Died
Pupae	26.48 ± 3.38	25.20 ± 2.43	23.39 ± 2.51	21.75 ± 2.01 *	20.22 ± 1.61 **
rAPSL	28.57 ± 2.81	25.15 ± 3.23	21.33 ± 3.10 **	19.09 ± 2.80 **	15.00 ± 2.72 ***

^▲▲▲^ *P* < 0.001 *vs.* control, * *P* < 0.05 *vs.* model, ** *P* < 0.01 *vs.* model, *** *P* < 0.001 *vs.* model, *x* ± s, *n* = 10–12.

**Figure 8:**
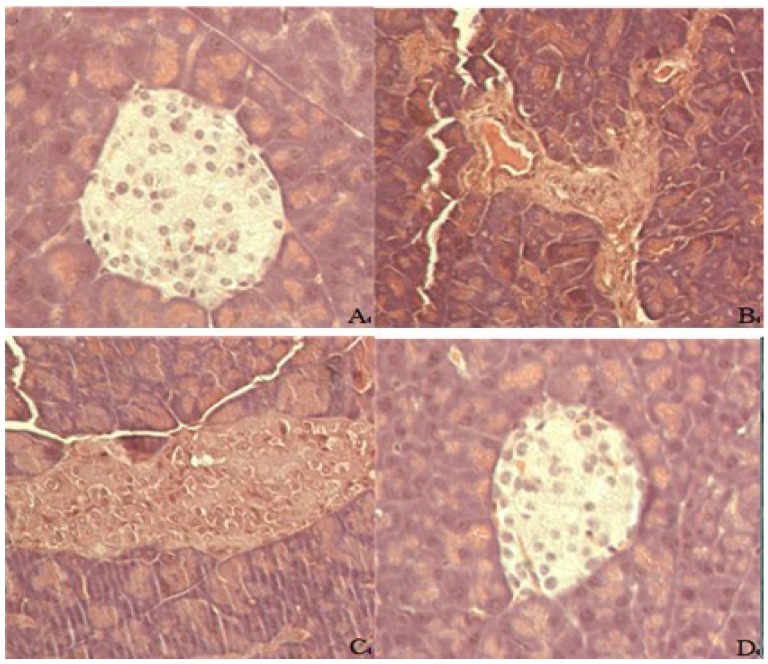
Histological analysis of mouse pancreatic tissues (200× HE). (**A**) Control; (**B**) model; (**C**) treated with freeze-dried powder from pupae not infected with rBv-*APSL*; (**D**) treated with rAPSL (10 mg/kg·day).

The analysis of paraffin sections of mouse pancreatic tissues indicated that rAPSL could effectively protect mouse islets from lesions induced by streptozotocin. As shown in Figure 8, the pancreatic tissues from the model mice exhibited islet atrophy, edge irregularities and disordered pancreatic acinar cells. However, the pancreatic tissues from rAPSL-treated mice exhibited regular edges and no distinct infiltration of the islet.

## 3. Discussion

In the Bac-to-Bac system, the donor plasmid pFastBac HTA containing the *APSL* gene was used to construct the rBacmid-*APSL*, which was then used to infect BmN cells to obtain rBv-*APSL*. The BmN cells, fifth instar larvae and diapausing pupae were infected with rBv-*APSL*. This allowed for rAPSL to be harvested using the baculovirus expression vector system (BEVS). Moreover, it is also safe for use in vertebrate animals [9,10]. *B. mori* has a well-studied genetic background and can be easily bred on a large scale. Additionally, it can also be utilized for high-capacity production at a low cost [22]. These features make the silkworm system an ideal expression and delivery package for producing medicinal proteins for oral delivery.

In this study, large-scale preparation of rAPSL was performed with genetic engineering and a silkworm bioreactor. A preliminary study of the oral absorption of rAPSL was also performed. Natural APSL could promote the repair of damaged islets and the secretion of insulin to treat diabetes. There are no related reports about the oral administration of rAPSL, so far.

Mice with streptozotocin-induced diabetes mellitus were treated with rAPSL for four weeks. The FBG levels were significantly decreased to 15.00 ± 2.72 mmol/L (*P* < 0.001 *vs.* model). The analysis of paraffin sections of mouse pancreatic tissues indicated that rAPSL could effectively protect mouse islets from streptozotocin-induced lesions. Early studies have also demonstrated that shark hepatic stimulator substance has protective effects against alloxan-induced diabetes in mice [23]. Therefore, rAPSL has promising prospects for the clinical treatment of type 2 diabetes mellitus. 

In modern society, diabetes mellitus and its related complications have become more common with the rapid development of technology and the increasing standard of living, particularly in developing countries. People spend less time exercising and more time working. This lifestyle coupled with an unhealthy and disordered diet has led to an increase of the number of people with diabetes mellitus. China is the largest developing country, and the incidence of diabetes has increased year after year. The increasing incidence of diabetes results in significant economic losses for individuals, families, health care systems and the nation [24,25]. Insulin is an effective diabetes drug, but it is not ideal. The existing drugs for diabetes have various side effects; worse still, they cannot effectively inhibit the exhaustion of islet cells. In this regard, rAPSL seems highly promising, as it is able to reduce the blood glucose level in mice with type 2 diabetes by repairing damaged islets and promoting the recovery of islets. Because the source of the natural shark liver peptide drugs is scarce, we have turned to genetic engineering and the silkworm bioreactor to produce large amounts of rAPSL for preliminary research on the oral administration of this peptide.

Our study demonstrates for the first time rAPSL synthesis using the silkworm baculovirus expression vector system. Sharks are marine organisms, and products derived from sharks have a low toxicity when orally administered to mammals [9,10]. Silkworms contain a large number of natural protease inhibitors, and the oral formulation that we prepared contained silkworm-expressed rAPSL, along with these protease inhibitors. The rAPSL was enclosed by lipidosome in the pupae, these protease inhibitors and lipidosome may have a positive effect on helping rAPSL to avoid the gastrointestinal digestive enzymes. Our work may pave the way for the development of an oral drug for the treatment of diabetes mellitus in the future.

## 4. Experimental

### 4.1. Materials

DNA manipulation and PCR amplification kits were purchased from TaKaRa Biomedicals (Kyoto, Japan). The Viral Genomic DNA Purification Kit was purchased from Roche Co. (San Francisco, CA, USA). The pFasBac HTA plasmid was purchased from Invitrogen. The Lipofectamine 2000 Reagent was purchased from Invitrogen (Carlsbad, CA, USA). The BmN cells, which originated from the ovary, were maintained in our laboratory and cultured at 27 °C in Sf-900 II Serum Free Medium Complete (GIBCO, Gran Island, NY, USA) containing 10% Fetal Bovine Serum (GIBCO, Gran Island, NY, USA). The *E. coli* DH10Bac/BmNPV was constructed and supplied by our laboratory. The fifth instar silkworm larvae and diapausing pupae, Jingsong × Haoyue, were reared under a photoperiod schedule of 12 h light and 12 h darkness at 25 ± 1 °C and provided from Zhejiang Chinagene Biomedical Co., Ltd. (Haining, China). Male ICR mice were provided by Hangzhou Normal University Animal Center (Hangzhou, China) and were housed at the central animal facility, where they were screened for bacterial and viral pathogens. Streptozotocin was purchased from Sigma Co. (St. Louis, MO, USA).

### 4.2. Amplification, Cloning and Identification of the APSL Gene

To obtain the target *APSL* fragment, primers for PCR amplification were designed as follows: forward primer 5′-GGAA*GGATCC*ACTGGTGGTGGGGCCGATCG-3′ (*Bam*H I) and reverse primer 5′-GGGC*GTCGAC*TTATCACCATTTAAAGGATTTC-3′ (*Sal* I). PCR was performed using the following conditions: 3 min at 94 °C, then cycled (30 cycles) for 30 s at 94 °C, 30 s at 55 °C and 45 s at 72 °C and finally held at 72 °C for 10 min. The PCR product were analyzed by 1.0% agarose gel electrophoresis and stored at −20 °C until use. 

### 4.3. Construction of the Recombinant Plasmid pFastBac HTA-APSL

The PCR product were digested with *BamH* I/*Sal* I and then cloned into the same cloning sites of pFastBac HTA using T4 DNA ligase. The recombinant transfer vector was designated pFastBac HTA-*APSL* and the ligation product was transformed into *E. coli* TG1 cells. After selecting the positive clones, the recombinant donor vector pFastBac HTA-*APSL* was obtained. The sequence of the inserted *APSL* gene synthesized by PCR was conﬁrmed by nucleotide sequencing.

### 4.4. Construction, Isolation and Analysis of the Recombinant Bacmid

Approximately 2 ng of recombinant donor plasmid was transformed into 200 µL of *E. coli* DH10Bac-competent cells, incubated at 37 °C for 4 h, serially diluted in SOC medium and spread evenly on plates containing antibiotics (50 μg/mL kanamycin, 7 μg/mL gentamicin and 10 μg/mL tetracycline), 100 μg/mL X-gal and 40 μg/mL isopropyl-β-d-thiogalactoside (IPTG). After incubation for 48 h at 37 °C, the white colonies (expected to represent recombinant BmNPV bacmids equipped with the *APSL* gene) were picked and cultured overnight in a medium containing kanamycin, gentamycin and tetracycline. 

The rBacmid-*APSL* was isolated from the DH10Bac cells using an AxyPrep Bacterial Genomic DNA Miniprep Kit (Axygen, CA, USA) and identified by PCR with the M13 forward primer (5′-GTTTTCCCAGTCACGAC-3′) and the M13 reverse primer (5′-CAGGAAACAGCTATGAC-3′). The PCR conditions were as follows: pre-denaturation at 93 °C for 3 min; 30 cycles at 94 °C for 45 s, 55 °C for 45 s, 72 °C for 5 min; and a final incubation at 72 °C for 10 min. The wild-type bacmid was used as the negative control group. The PCR product was analyzed by 1.0% agarose gel electrophoresis. The recombinant bacmid was named rBacmid-*APSL*.

### 4.5. Expression of the APSL Gene in BmN Cells

To generate the recombinant baculovirus, the rBacmid-*APSL* was transfected into BmN cells using the Lipofectamine 2000 Reagent (Invitrogen, Carlsbad, CA, USA). Five days after transfection, the medium supernatant was collected and used to infect the BmN cells, according to the manufacturer’s protocol. The recombinant virus (rBv-*APSL*) was then prepared, and the titer of the virus was calculated using the Reed-Muench method. The genome of rBv-*APSL* was obtained with the Viral Genomic DNA Purification Kit (Axygen, Union City, CA, USA) and analyzed by PCR with P1 and P2, P1 and the M13 reverse primers, the M13 forward and P2 and the M13 forward and M13 reverse primers. The PCR conditions for the P1 and P2 primers were the same as the PCR conditions for the pFastBac HTA-*APSL*. The conditions for PCR analysis with the other three primer pairs were the same as the conditions for the PCR analysis of rBacmid-*APSL*. 

BmN cells in a 35 mm dish (containing approximately 4 × 10^6^ cells) were infected with recombinant virus at a MOI of 10. The infected cells were cultured at 27 °C, harvested 120 h after infection and the harvested cells were resuspended in 0.2 mL of phosphate buffered saline (PBS) before being disrupted using gentle sonication for 5 min on ice. The samples were stored at −80 °C after centrifugation until further analysis. Then the cells lysis supernatant was subjected to 12% SDS-PAGE. The separated protein bands were transferred to a NC ﬁlter membrane. The membrane was blocked for 2 h in Tris-buffered saline (TBS) containing 5% nonfat dry milk and incubated with antibodies for APSL (Rabbit anti-APSL was produced by us). After it was washed in Tris-buffered saline with Tween (TBST, 0.5% Tween-20), the corresponding secondary antibodies were incubated for 2 h. Detection of the immunoreaction was performed with a 3,3′-diaminobenzidine (DAB) Kit (CWBIO, Beijing, China).

### 4.6. Expression of the APSL Gene in Silkworm Larvae and Pupae

Fifth instar silkworm larvae and diapausing pupae were infected with rBv-*APSL* by subcutaneous injection. A significant incidence of the silkworm larvae and pupae appeared from 72 h to 120 h post-infection. Hemolymph from the infected larvae was collected at 144 h post-inoculation and centrifuged at 12,000× *g* for 30 min at 4 °C to remove the insoluble impurities. The hemolymph samples were then stored at −80 °C. The infected pupae were collected at 144 h post-inoculation and crushed. The pupal mash was centrifuged at 12,000× *g* for 30 min at 4 °C to remove most of the top lipids layer and the bottom layer of debris. The upper solution was centrifuged three times as described above to remove the remaining lipids and debris. The pupal supernatant was stored at −80 °C. The hemolymph from the silkworm larvae and pupae was freeze-dried, and the powder was subjected to 12% SDS-PAGE. The separated protein bands were transferred to a NC ﬁlter membrane and analyzed by Western blotting, as previously described in Section 4.5. 

### 4.7. Protective Effects against Streptozotocin-Induced Diabetes Mellitus in Mice

Forty-eight ICR male mice, weighing 18–22 g, were randomly placed in the following four groups: the control group, the model group, the silkworm pupae-treated group and the rAPSL-treated group. The last three groups were fed a high-sugar and high-fat diet for four weeks. To induce diabetesmellitus, all the mice except for the control group were injected with streptozotocin (80 mg/kg body wt) intraperitoneally 12 h after fasting. The FBG levels were measured by Roche glucometer (Accu-Chek Advantage, Mannheim, Germany) 72 h after streptozotocin administration. Only the mice with FBG levels over 25 mmol/L were selected for the experiments. The mice in the control and model groups were treated with 0.15 M PBS (0.2 mL/10 g wt) only, the mice in the rAPSL-treaded group were treated with rAPSL (10 mg/kg·day) via intragastric administration and the mice in the silkworm pupae-treated group were treated with freeze-dried powder from pupae not infected with rBv-*APSL* via intragastric administration. The FBG levels were measured again on the 10th, 17th, 24th and 31th day using a glucometer after streptozotocin administration.

At the end of the fifth week, all the mice were executed, and pancreases were fixed in 10% neutral-buffered formalin for two weeks, followed by processing for conventional paraffin embedding. Sections (8 μm) were mounted on glass slides, dewaxed in xylene, rehydrated through graded alcohols, washed in distilled water and stained with hematoxylin and eosin (HE). All slides were examined under a microscope.

### 4.8. Statistical Analysis

Data are presented as the mean ± standard deviation. Statistical analysis of the data for multiple comparisons was performed by analysis of variance (ANOVA). For single comparison, the significance of differences between means was determined by *t*-test. Values of *P* < 0.05 were considered statistically significant, and a value of *P* < 0.001 was considered statistically most significant.

## 5. Conclusions

In this study, rAPSL was expressed by rBv-*APSL* using the Bac-to-Bac baculovirus expression system. rBv-*APSL* successfully infected silkworm pupae, and the infected silkworm pupae were used as a bioreactor to express rAPSL. Studies have shown that rAPSL has a good effect of anti-type 2 diabetes in type 2 diabetic mice and effectively protects mouse islets from streptozotocin-induced lesions. The results of this study could be useful in the industrialization of rAPSL and the development of a new type anti-type 2 diabetic oral drugs in further research.
